# Genetic diversity and association analysis of the growth hormone gene in local Indonesian chicken

**DOI:** 10.5455/javar.2025.l976

**Published:** 2025-12-25

**Authors:** Nurul Pratiwi, Nurul Azizah, Anne Sukmara, Bayu Dewantoro Putro Soewandi, Latifah Latifah, Tuti Haryati, Bram Brahmantiyo, Jonathan Anugrah Lase, Dwi Rohmadi, Usman Usman, Paskah Partogi Agung, Maijon Purba, Mohamad Farid Ridhillah, Abdullah Baharun, Ferdy Saputra, Tike Sartika

**Affiliations:** 1 Research Center for Animal Husbandry, National Research and Innovation Agency of the Republic of Indonesia (BRIN), Cibinong, Bogor, Indonesia.; 2 Directorate of Laboratory Management, Research Facilities, and Science and Technology Park, National Research and Innovation Agency of the Republic of Indonesia (BRIN), Bogor, Indonesia.; 3 Research Center for Applied Zoology, National Research and Innovation Agency of the Republic of Indonesia (BRIN), Bogor, Indonesia.; 4 Department of Animal Science, Faculty of Agriculture, Universitas Djuanda, Bogor, Indonesia.

**Keywords:** Carcass traits, genetic diversity, growth hormone, Indonesian chickens, laying traits

## Abstract

**Objective:**

The aim of this study was to investigate the effects of theGrowth hormonegene, including its additive and dominant components, on laying performance (age at first laying, body weight at first laying, weight of the first egg, total eggs produced by 24 weeks, and total eggs produced by 48 weeks) and carcass attributes (live weight, carcass weight, breast weight, back weight, thigh weight, and wing weight).

**Materials and Methods:**

A total of 35 Kampung hens and 19 Sentul roosters were used in this study. Genotyping at the GH|MspI and GH|SacI loci was conducted using the restriction fragment length polymorphism (RFLP) method. Association analyses were conducted using analysis of variance, while additive and dominance effects were assessed through generalized linear models implemented in R version 4.3.1.

**Results:**

RFLP analysis revealed polymorphism at the GH|MspI locus but not at GH|SacI. No significant differences in any trait were observed among the GH|MspI genotypes. Similarly, no significant additive or dominance effects were detected. Moreover, nanopore sequencing identified two novel single-nucleotide polymorphisms, g.3242delG and g.3396_3401del, in both Kampung and Sentul chickens.

**Conclusion:**

No significant association was found between these polymorphisms and laying or carcass traits. Additionally, no significant additive or dominance effects were found, implying that these loci did not significantly influence the definition of these traits in Kampung and Sentul chickens.

## Introduction

The local chicken breed in Indonesia is considered a slow-growing breed that thrives well in tropical climates. Nonetheless, the adaptability of birds of this breed to tropical environments resulted in resistance to viruses during the outbreak of Avian Influenza (AI) in Indonesia [1], thus showing the potential to meet the demand for chicken meat and eggs. Kampung chickens of Indonesia are laying chickens that can yield up to 103 eggs within 24 weeks [2]. On the other hand, Sentul chickens are a native breed from Ciamis, typically reared for meat production at around 10–12 weeks of age [3]. These two local chicken breeds are being considered for commercialization within the community, and yet a more thorough exploration of their genetic diversity for quality selection is necessary.

Genetic variation in Indonesian chickens has been assessed by several studies using microsatellites [4,5], mitochondrial DNA (Deoxyribonucleic Acid) [6-8], and functional genes [9]. Additionally, single-nucleotide polymorphisms (SNPs) add value for breeding and improve the efficiency of marker-assisted selection [10,11]. Chicken genes that regulate growth hormones are crucial in controlling the rate of growth, development, metabolism, and numerous other physiological processes. Growth hormone (GH) plays a crucial role in regulating protein, lipid, and carbohydrate metabolism, as well as influencing growth, development, and the immune system’s functioning [12]. Chickens are widely used as poultry, and understanding the structure, function, and regulation of the growth hormone gene is crucial for optimizing growth and production in these birds. In chickens, the* GH* gene is located on chromosome 27 and spans five exons, ~3,507 base pairs (bp). As a signaling molecule, this gene regulates the proliferation and division of target cells by modulating the transcriptional activity of genes involved in cell growth and tissue repair [13].

GH polymorphisms have been associated with growth and carcass composition in several breeds [14] and have been explored as candidate markers for egg production [15]. Therefore, understanding the role of the* GH* gene is essential for improving the productivity of local Indonesian chickens. This study aimed to analyze the association between the* GH* gene locus and laying as well as carcass traits, considering both additive and dominance genetic effects.

## Materials and Methods

### Ethical approval

All experiments involving chickens were conducted in accordance with the ethical guidelines approved by the Division of Animal Care and Use—Ethical Clearance and Research Permit Commission, National Research and Innovation Agency (BRIN), Indonesia (Approval No. 211/KE.02/SK/12/2023; date: 05 December 2023).

### Chickens

A total of 35 female Kampung chickens were assessed for production traits, including age and body weight at first lay, initial egg weight, and the number of eggs produced up to 24 and 48 weeks of age. Additionally, 19 male Sentul chickens were slaughtered at 10 weeks for carcass evaluation. A 3 ml blood sample was collected from the brachial vein and transferred to Vacutainer tubes containing EDTA as an anticoagulant.

### DNA extraction and polymerase chain reaction

The Quick-DNA Miniprep Kit (Zymo Research, USA) was used for genomic DNA extraction, and* GH* gene primers and restriction enzymes were used as described by Kuhnlein et al. [16]. The primer ofthe* GH* gene was a forward primer (5’-CTA AAG GAC CTG GAA GAA GGG-3’) and a reverse primer (5’-AAC TTG TCG TAG GTG GGT CTG-3’); the amplicon length was 1,164 bp (base pairs). Each reaction volume (15 μl) was prepared to contain, in that order, 1 μl of sample DNA, 5.5 μl of DNA/RNA-free water, 0.5 μl of forward primer, 0.5 μl of reverse primer, and 7.5 μl of MyTaq HS Red Mix (Bioline, UK). The conditions of the reaction were carried out under the following conditions: the initial denaturation was at 95°C for 5 min, followed by 35 cycles: denaturation at 95°C for 30 sec, annealing at 66°C for 30 sec, extension at 72°C for 30 sec, and a final extension at 72°C for 5 min. Restriction fragment length polymorphism (RFLP) was used for genotyping. The restriction digest was prepared by mixing 1.0 μl buffer, 1.0 μl restriction enzymes (MspI and SacI), 5.0 μl amplicon, and 9.0 μl DNA/RNA-free water, then incubating the mixture at 37°C for 2 h. Each digestion product (5 μl) was separated on a 2% agarose gel at 100 V for 35 min with a 100 bp DNA ladder as a size marker, and the DNA fragments were visualized under UV illumination.

### Amplicon sequencing using nanopore

Amplicon sequencing was conducted using the MinION platform equipped with a Flow Cell R10.4.1 and the Native Barcoding Kit 24 V14 (Oxford Nanopore Technologies, UK). Sequencing adapters were trimmed with Porechop [17], and read alignment was performed using minimap2 [18]. Consensus sequences were subsequently constructed with the iVar tool [19].

### Statistical analysis

POPGEN version 1.32 was used to obtain allele frequencies, as well as observed and expected heterozygosity [20]. Polymorphism information content was obtained using the Cervus version 3.07 [21]. The data analysis was conducted using R 4.3.1 [22]. Following a significant analysis of variance, the mean values were compared using the Tukey–Kramer HSD post hoc test. The statistical model for the association of* GH* genotype was

*Y*_*ijk*_ = μ + *g*_*j*_ + ε_*ijk*_

where *Y*_*ijk*_ was the response variable, μ was the population mean, *g*_*j*_ was the genotype effect, i was the index for individuals, *j* was the index for genotype, and *k* was the index for the response variable. ε_*ijk*_ was the random error.

An examination of the additive and dominant effects was carried out using the generalized linear model:

*y* = μ + *C*_*aa*_ + *C*_*dd*_ + *e*

where *y* was the response variable of each phenotype; μ was the intercept; C_*aa*_ was a covariate coefficient with additive effect (a); and C_*dd*_ was a covariate coefficient with dominance effect (d); e was the residual standard error.

### Results

### Genetic diversity of GH|MspI and GH|SacI in Kampung and Sentul chicken

**Figure 1.**
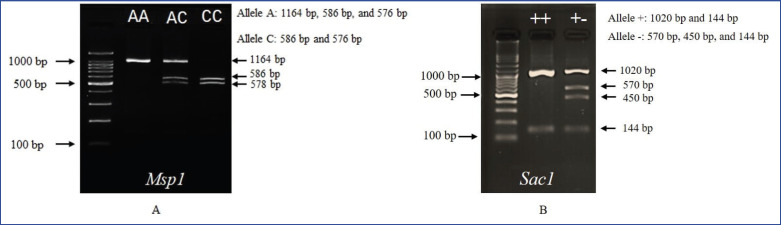
Genotyping of GH|MspI (A) and GH|SacI (B) in Kampung and Sentul chickens.

The RFLP analysis of Kampung and Sentul chickens in this study revealed that GH|MspI had two alleles and three genotypes, whereas GH|SacI had a single allele and one genotype (Fig. 1). Figure 1 showed the agarose gel electrophoresis results for* GH* gene polymorphisms using the MspI and SacI restriction enzymes. Panel (A) represented the GH|MspI digestion, where three genotypes were observed: AA, AC, and CC. The AA genotype showed a single band of 1164 bp (base pairs), and the AC genotype showed three bands (1164 bp, 682 bp, and 482 bp), indicating heterozygosity. The CC genotype showed two bands (682 bp and 482 bp), representing complete digestion. Panel (B) illustrated the GH|SacI digestion, where only one genotype (++) was present. The digestion showed two bands at 1,026 bp and 138 bp, implying that there is no polymorphism at this locus.

Table 1 showed genotype frequency, allele frequency, observed (Ho) and expected (He) heterozygosities, and polymorphism information content (PIC). In Sentul and Kampung chickens, the AA genotype at the GH|MspI locus was dominant, occurring at frequencies of 0.684 and 0.543, respectively. Moreover, the A allele was found to be more dominant among Kampung and Sentul chickens, with frequencies of 0.729 and 0.816, respectively. The heterozygosity values also confirm that Kampung chickens have higher heterozygosity compared to Sentul chickens, indicating a greater genetic diversity in the population of Kampung chickens. The genotype frequency calculated at the GHidSacI locus of Kampung chicken was 1.000, with both the Kampung and Sentul chickens having the same genotype of ++. The PIC value analysis revealed that GH|MspI had a higher value than GH|SacI, indicating that the GH|MspI marker is more effective for identifying genetic differentiation.

**Table 1.** Summary statistic of genotype frequency, allele frequency, observed (Ho), and expected (He) heterozygosities, and PIC.

Loci	Breed	Genotype frequency			Allele frequency		Ho	He	PIC
		AA	AC	CC	A	C			
GH|MspI	Kampung	0.543	0.371	0.086	0.729	0.271	0.371	0.401	0.299
	Sentul	0.684	0.263	0.053	0.816	0.184	0.263	0.308	
		++	+-	--	+	−			
GH|SacI	Kampung	1.000	0.000	0.000	1.000	0.000	0.000	0.000	0.000
	Sentul	1.000	0.000	0.000	1.000	0.000	0.000	0.000	

Association of GH|MspI and GH|SacI in Kampung and Sentul Chickens: Table 2 presented the association of GH|MspI and GH|SacI in Kampung chickens. For the laying characteristics of Kampung chickens, the analysis revealed that the traits were not statistically affected by GH|MspI. The AC genotype had an earlier age at first egg laying by a few weeks and, consequently, showed slightly higher egg production at 24 and 48 weeks compared to other genotypes.

**Table 2:** The association of growth gene polymorphism with laying traits of Kampung chicken.

Traits	GH|MspI				GH|SacI
	Genotype				Genotype
	AA ( *n* = 19)	AC ( *n* = 13)	CC ( *n* = 3)	*p* -value	++ ( *n* = 35)
AFEL (days)	147.78 ± 16.85	139.23 ± 7.99	141.33 ± 7.09	0.22	144.05 ± 13.05
BWFL (gm)	1,764.84 ± 183.42	1,764.00 ± 183.42	1,871.00 ± 99.95	0.21	1,733.34 ± 184.37
EWFL (gm)	31.21 ± 6.45	29.00 ± 2.88	28.33 ± 3.05	0.42	30.14 ± 5.19
EN24 (eggs)	107.26 ± 35.16	108.15 ± 21.79	95.33 ± 49.09	0.81	106.57 ± 31.24
EN48 (eggs)	196.52 ± 70.38	197.15 ± 56.23	193.00 ± 81.41	0.99	196.45 ± 64.26

AFEL: age at first egg laying; BWFL: body weight at first egg laying; EWFL: egg weight at first laying; EN24: egg number from onset of laying eggs to 24 weeks; EN48: egg number from the onset of laying eggs to 48 weeks.

The association study of GH|MspI and GH|SacI in Sentul chickens is presented in Table 3. Statistical analysis showed that the GH|MspI locus had no significant effect on carcass traits in Sentul chickens. However, the AC genotype had a slightly higher breast weight than any other genotype, and the CC genotype had higher thigh and wing weights. These results suggest that GH|MspI may not be a significant statistical factor in average carcass characteristics; however, some genotypes may still contribute to variation in individual patterns of meat distribution. Table 4 summarizes the additive and dominance effects on laying and carcass traits. It was statistically found that the additive and dominance effects were insignificant on the laying traits in Kampung chicken, indicating that the genetic differences at the examined loci do not significantly affect the egg production-related characteristics of this breed. Similarly, there were no significant differences in carcass traits in Sentul chickens, regardless of whether additive effects or dominance effects were considered. This indicates that A and C alleles at a tested locus do not cause any detectable variation in carcass traits in this breed. On the whole, these results suggest thatthe* GH* MspI and* GH* SacI polymorphs examined in this work might not have a strong effect on laying performance or carcass quality, at least in the populations considered.

**Table 3.** The association of growth gene polymorphism with carcass traits of Sentul chicken.

Traits	GH|MspI				GH|SacI
	Genotype				Genotype
	AA ( *n* = 13)	AC ( *n* = 5)	CC ( *n* = 1)	*p* -value	++ ( *n* = 19)
Live weight (gm)	1,070.38 ± 100.07	1,176.20 ± 98.95	1,210	0.10	1,105.57 ± 108.34
Carcass weight (gm)	707.69 ± 75.91	747.40 ± 79.01	753.00	0.55	720.52 ± 72.78
Breast weight (gm)	178.23 ± 31.01	198 ± 21.76	189	0.44	184 ± 28.74
Back weight (gm)	176 ± 19.48	181.40 ± 9.28	182.50	0.81	177.76 ± 16.72
Thigh weight (gm)	223.43 ± 64.99	211.26 ± 106.63	261.30	0.83	222.22 ± 73.90
Wing weight (gm)	111.84 ± 11.65	112.40 ± 7.66	119.70	0.78	112.41 ± 10.33

**Table 4.** The additive and dominance effect of GH|MspI with laying traits and carcass traits.

Kampung				
Traits	Additive	*p* -value	Dominance	*p* -value
AFEL	−2.1 ± 8.75	0.81	10.66 ± 11.38	0.35
BWFL	−106.15 ± 116.05	0.36	31.36 ± 150.90	0.83
EWFL	0.67 ± 3.33	0.84	1.52 ± 4.34	0.72
EN24	12.82 ± 20.50	0.53	−13.71 ± 26.66	0.61
EN48	4.78 ± 42.42	0.92	−4.78 ± 55.15	0.93
Sentul				
Live weight	−33.80 ± 109.32	0.76	−72.02 ± 136.72	0.60
Carcass weight	−5.60 ± 81.50	0.94	−34.11 ± 101.92	0.74
Breast weight	9.00 ± 31.75	0.78	−28.77 ± 39.71	0.47
Back weight	−1.10 ± 19.18	0.95	−4.30 ± 23.98	0.86
Thigh weight	−50.04 ± 84.93	0.56	62.21 ± 106.22	0.56

AFEL: age at first egg laying; BWFL: body weight at first egg laying; EWFL: egg weight at first laying; EN24: egg number from the onset of laying eggs to 24 weeks; EN48: egg number from the onset of laying eggs to 48 weeks.

*p* < 0.05 indicates statistically significant differences.

### Single-nucleotide polymorphisms in the growth hormone gene (intron 4)

A study of polymorphism in growth hormone genes in Kampung and Sentul chickens revealed that only MspI, one of the nine enzymes examined, was found to be present in these chickens. Thus, amplicon sequencing was done using Nanopore to estimate genetic diversity in the Intron 4 region. In this study, 37 SNPs were observed, of which g.3242delG and g.3396_3401del were two new mutations recorded in Kampung and Sentul chickens (Table 5). The remaining 34 mutations were identified as commonly occurring based on existing databases and references.

**Table 5.** Genetic variation of the growth hormone gene in Intron 4.

SNPs*	Restriction enzyme	Breed
g.2661A>G	CviQI	Kampung, Sentul
g.2685C>A	Hpy188I	Kampung
g.2731G>A	None	Kampung, Sentul
g.2774T>C	None	Kampung, Sentul
g.2807C>T	None	Kampung
g.2809G>A	None	Kampung, Sentul
g.2831C>T	BtsCI	Kampung, Sentul
g.2880G>A	None	Kampung
g.2965T>C	Fnu4HI	Kampung
g.3018G>A	None	Kampung, Sentul
g.3029A>G	None	Kampung, Sentul
g.3094T>C	MspI	Kampung, Sentul
g.3113C>T	None	Kampung, Sentul
g.3126T>C	None	Sentul
g.3127C>T	None	Kampung, Sentul
g.3129A>T	None	Kampung, Sentul
g.3242delG^#^	None	Kampung, Sentul
g.3245C>T	None	Kampung, Sentul
g.3261C>T	None	Kampung
g.3267C>T	None	Kampung
g.3267G>A	FatI	Kampung, Sentul
g.3318G>A	None	Kampung, Sentul
g.3373C>T	Nt.CviPII	Kampung
g.3384T>C	None	Kampung, Sentul
g.3396_3401del^#^	DpnI	Kampung, Sentul
g.3412C>A	None	Kampung, Sentul
g.3425C>T	None	Kampung, Sentul
g.3451G>T	Alu1	Sentul
g.3455C>A	None	Kampung, Sentul
g.3481A>G	None	Kampung, Sentul
g.3500G>A	HinP1I	Kampung, Sentul
g.3515C>T	None	Kampung
g.3519A>G	HinP1I	Kampung, Sentul
g.3521G>A	None	Kampung, Sentul
g.3581G>T	BstUI	Kampung, Sentul
g.3641T>C	None	Kampung, Sentul
g.3715A>G	AciI	Kampung, Sentul

*Position based on Genbank access number AY461843.1, ^#^: New mutations

## Discussion

Growth hormone gene polymorphism analysis of Kampung and Sentul chickens showed that the GH|MspI locus had two alleles (A and C) and three genotypes (AA, AC, and CC), which genetically varied, and the GH|SacI locus had one genotype (++), which was not polymorphic. Although the GH|MspI marker proved to be informative of genetic variation, the association analysis did not show statistically significant effects of GH|MspI or GH|SacI genotype on laying traits in Kampung chickens or carcass traits in Sentul chickens.

Nonetheless, intron 4* GH* gene sequencing using Nanopore technology identified 37 SNPs, including two new deletions (g.3242delG and g.3396_3401del), which have not been previously recognized and could potentially cause variation in gene regulation. A major weakness of this study is that the sample sizes for the two chicken populations (35 Kampung and 19 Sentul) were relatively small and unequal, which diminished the statistical power to detect modest to small genetic effects. The post-hoc power analysis revealed that the study was underpowered (β = 0.82), suggesting that small associations might have been overlooked. Moreover, the GH|SacI locus was monomorphic in all individuals; thus, its use in association tests was nullified, and more informative markers were required.

The 22-kDa polypeptide GH is synthesized and secreted by eosinophilic cells within the anterior pituitary [23]. This hormone is crucial in regulating body weight, as it influences fat metabolism and promotes a higher feed efficiency ratio. In addition to its key role in growth,* GH* also influences reproductive traits in chickens. Su et al. [15] and Feng et al. [24] found that the* GH* gene influences egg production and the age of first egg-laying in White Leghorn chickens. These results highlight the extended physiological significance of GH, extending beyond growth, and its role in influencing growth performance and reproductive efficiency in poultry.

The researcher determines that the GH|MspI gene has three genotypes and two alleles, which means that genetic variability exists at this locus. Inthe* GH* gene intron 4, however, there should be three alleles, resulting in six genotypes [25,26], which suggests that the GH|MspI locus may harbor more genetic variations. Of particular interest is the role of intron 4 in gene regulation, where intronic differences may affect gene expression, which in turn can influence growth and metabolic characteristics in chickens.

Additional evidence was reported by Shafey et al. [27], who identified two novel point mutations, T77C and C485T, within the fourth intron of the* GH* gene. Intronic SNPs may hold biological significance by altering mRNA processing, stability, or regulatory pathways, which can, in turn, impact growth and metabolic efficiency in chickens.

GH|MspI polymorphisms have a functional effect, which can be observed in various breeds of chickens. According to Alfano et al. [28], the ++ genotype of the GH|MspI locus is better in body weight, weight gain, and morphometric characteristics than other genotypes in Kampung chicken. Likewise, Pratama et al. [29] reported that in Sentul chickens, the ++ genotype was closely associated with body weight and body weight gain, supporting the use of this genetic marker in improving growth performance.

Conversely, a study of local Egyptian chickens conducted by Mansour et al. [30] showed that carriers with the AA genotype of GH|MspI obtained lower abdominal fat compared to other genotypes. This indicates that GH|MspI polymorphisms affect both growth traits, although they may affect fat deposition and metabolic efficiency, which would have implications in broiler breeding and fat-cutting selection programs. Although certain associations were detected, the findings indicate that the GH|MspI locus has no significant influence on egg-laying performance or carcass characteristics. The above result suggests that although GH gene polymorphisms affect growth and fat metabolism, they may not be significant factors influencing reproductive performance or meat quality in chickens.

Genetic diversity in the GH|SacI locus is relatively low in Kampung and Sentul chicken species, with the allele + being more commonly prevalent. This suggests limited polymorphism at this site in these indigenous chicken breeds. Despite the low variability, Makhsous et al. [25] reported that the GH|SacI locus is associated with egg production, indicating that even minor genetic differences at this locus may influence reproductive performance. The GH|SacI polymorphism resides within intron 4 of the* GH* gene. Notably, previous studies have associated this polymorphism with disease susceptibility. Liu et al. [31] and Kuhnlein et al. [16] reported a relationship between this locus and the extent of tumor-affected tissues in White Leghorn chickens infected with Marek’s disease virus. These findings indicate that the GH|SacI site may influence not only production performance but also immune function and resistance to disease.

Beyond GH|SacI, another important polymorphism in the* GH* gene is SNP G+1705A, which has been linked to significant growth-related quantitative traits. This SNP can be specifically targeted by the EcoRV restriction enzyme [32,33]. The GH|EcoRV locus has been widely studied for its impact on growth performance. According to Anh et al. [14], the G allele of the GH|EcoRV locus is known to significantly influence body weight, average daily gain, and dressing percentage, with the G allele contributing positively to these traits.

Furthermore, Mariandayani et al. [34] provided additional evidence supporting the significance of GH|EcoRV in growth performance. Their findings indicated that this locus significantly influenced body weight, weight gain, feeding intake, and conversion efficiency over a 12-week period.

We found no additive or dominance effects for laying (AFEL, BWFL, EWFL, EN24, and EN48) and carcass traits (live weight, carcass weight, breast weight, back weight, and thigh weight).

In general, traits governed primarily by additive genetic effects can be improved more efficiently than those influenced by substantial non-additive effects [35]. In another paper, Lopes et al. [36] argued that the dominance effects have increased the rate of advancement more than considering only additive effects. Ignoring these dominance effects, however, can lead to a further deceleration in potential genetic progress. In the case of quantitative traits, Yang et al. [37] identify dominance effects as a factor contributing to variation in quantitative traits, including growth traits in farm animals.

Two previously undetected alleles were identified in Intron 4 of the Chinese indigenous breeds by Nie et al. [38]. Allele E is distinguished from allele D by the deletion of 50 base pairs: allele D features two MspI restriction sites. We identified a single MspI site as well as two novel mutations, g.3242delG and g.3396_3401del, in this research. Kulibaba et al. [39] additionally identified a novel AluI restriction site mutation within intron 4, which has been associated with variations in egg production, egg weight, live body weight, and carcass weight. Our research has revealed the occurrence of the AluI mutation in Sentul chickens, a breed identified as meat-type based on sequencing results. This mutation may be used as a selection marker. Further studies are needed with larger and more balanced cohorts and with a genome-wide SNP panel or sequencing studies to more fully capture genetic variation and discover associations with higher statistical power.

## Conclusion

We identified two novel intron 4 variants (g.3242delG, g.3396_3401del). Although they were not associated with the measured traits here, they expand the known* GH* variation in Indonesian chickens and warrant validation in larger, multi-breed cohorts.

## List of abbreviations

AA Genotype; AC Genotype; AFEL, age at first egg laying; BWFL, body weight at first egg laying; C, CC Genotype; DNA, deoxyribonucleic acid; EWFL, egg weight at first laying; EN24, egg number from the onset of laying eggs for 24 weeks; EN48, egg number from the onset of laying eggs to 48 weeks HSD, Tukey’s Honestly Significant Difference; RNA, Ribonucleic Acid.
